# Biological and Metabolic Correlates of Psychological Resilience in Maintenance Hemodialysis: An Exploratory Gender-Based Study

**DOI:** 10.3390/medicina62061207

**Published:** 2026-06-22

**Authors:** Gloria María Zaragoza Fernández, Avinash Chandu Nanwani, Elena Jiménez Mayor, Celia Rodríguez Tudero, Esperanza Moral Berrio, Alonso González de Gregorio, Marco Vaca Gallardo, Enrique Antonio Florit, José C. De La Flor, Rafael Fernández Castillo

**Affiliations:** 1 Department of Nephrology, Central Defense Hospital Gómez Ulla, 28047 Madrid, Spain; jflomer@mde.es; 2 Department of Nephrology, Hospital General de Fuerteventura, 35600 Fuerteventura, Spain; achanan@gobiernodecanarias.org; 3 Department of Nephrology, Hospital Santa Bárbara, 42003 Soria, Spain; ejimenezmay@saludcastillayleon.es; 4 Department of Nephrology, Hospital Universitario de Salamanca, 37007 Salamanca, Spain; crodrigueztudero@usal.es; 5 Surgery Department, Faculty of Medicine, University of Salamanca, 37007 Salamanca, Spain; 6 Department of Nephrology, Hospital General Universitario de Ciudad Real, 13005 Ciudad Real, Spain; emoral@sescam.jccm.es; 7 Psychology Area, True Horizon Consulting, 08034 Barcelona, Spain; alonso.glezdegregorio@gmail.com; 8 Department of Nephrology, Hospital Universitario Gregorio Marañon, 28007 Madrid, Spain; marco.vaca@salud.madrid.org; 9 Clinic Institute of Nephrology and Urology, Hospital Clinic, 08036 Barcelona, Spain; eflorit@sistemes-renales.com; 10 Department of Medicine and Medical Specialties, Faculty of Medicine, Alcala University, 28805 Madrid, Spain; 11 Biosanitary Research Institute of Granada (ibs.GRANADA), 18010 Granada, Spain; rafaelfernandez@ugr.es; 12 Nursing Department, University of Granada, 18012 Granada, Spain

**Keywords:** resilience, gender, hemodialysis, β_2_-microglobulin, serum albumin

## Abstract

*Background and Objectives*: Psychological resilience is central to emotional adaptation in patients undergoing maintenance hemodialysis (HD). Although psychosocial determinants have been widely studied, the role of routinely monitored biochemical markers remains insufficiently defined. *Materials and Methods*: This study examined the associations between selected metabolic–inflammatory biomarkers and psychological resilience in adults receiving maintenance HD and explored potential gender-related differences. Resilience was assessed using the Resilience Scale–14 (RS-14). β_2_-microglobulin, serum albumin, calcium, and 25-hydroxyvitamin D were analyzed as continuous predictors. Multiple linear regression models with heteroscedasticity-consistent robust standard errors (HC3) were adjusted for age, HD vintage, diabetes, and cardiovascular disease. Two interaction terms (Gender × β_2_-microglobulin and Gender × albumin) were specified a priori. Model stability was evaluated using nonparametric bootstrap resampling (5000 iterations) and penalized regression with cross-validation. *Results:* In bivariate analyses, higher β_2_-microglobulin levels were associated with lower resilience (ρ = −0.24; *p* = 0.041), whereas serum albumin showed a positive but non-significant association (*p* = 0.14). These relationships did not remain statistically significant in fully adjusted models (β_2_-microglobulin: *p* = 0.107). No Gender × Biomarker interaction reached statistical significance (*p* = 0.162). Stratified analyses showed consistent directional patterns across gender groups. *Conclusions:* Metabolic–inflammatory biomarkers, particularly β_2_-microglobulin and serum albumin, may be associated with psychological resilience in HD. However, gender-specific effects were not supported in adjusted analyses. These findings require validation in larger, longitudinal, multicenter studies.

## 1. Introduction

End-stage kidney disease (ESKD) represents a major clinical and psychosocial burden characterized by irreversible loss of kidney function and dependence on renal replacement therapies such as maintenance hemodialysis (HD). Despite therapeutic advances, patients receiving HD continue to experience high morbidity, impaired quality of life, frequent emotional distress, and substantial variability in psychological adaptation to chronic illness [1,2].

Within this context, psychological resilience—defined as the capacity to maintain or regain emotional and functional stability in the face of adversity—has emerged as a clinically relevant determinant of adaptive outcomes in HD populations. Higher resilience has been associated with lower emotional distress, better coping strategies, improved treatment adherence, and greater quality of life among individuals undergoing maintenance HD [3,4,5,6,7,8,9]. Current evidence suggests that resilience is influenced predominantly by psychosocial factors, including social support, family dynamics, and coping resources, although the mechanisms underlying individual variability in resilience remain incompletely understood.

Previous studies have identified social support, self-efficacy, family resilience, and adaptive coping as important determinants of resilience in patients receiving maintenance HD, supporting the view of resilience as a dynamic and potentially modifiable process with relevance for patient-centered care [10,11,12,13].

Beyond psychosocial determinants, biological and metabolic factors may also contribute to adaptation in chronic dialysis settings. In routine HD care, biomarkers such as β_2_-microglobulin, serum albumin, calcium, and vitamin D are regularly monitored because they reflect inflammatory burden, nutritional status, and mineral metabolism, all of which have been associated with morbidity and clinical instability in HD populations [1,14,15,16,17,18,19]. However, the potential relationship between these routinely monitored biomarkers and psychological resilience has received limited attention.

In addition, gender may influence emotional adaptation, perceived treatment burden, coping strategies, and psychosocial vulnerability in individuals receiving maintenance HD [20,21,22]. Integrating a gender-sensitive perspective into resilience research may therefore contribute to a more comprehensive biopsychosocial understanding of adaptation in chronic dialysis care and support more individualized clinical approaches.

Against this background, the present exploratory study aimed to examine the associations between selected inflammatory and nutritional biomarkers (β_2_-microglobulin, albumin, calcium, and vitamin D) and psychological resilience in adults undergoing maintenance HD, and to explore whether these associations differed according to gender.

## 2. Materials and Methods

### 2.1. Study Design and Population

We conducted an observational, cross-sectional, single-center study in the Hemodialysis Unit of the Central Defense Hospital “Gómez Ulla” between January and June 2024. The study was conducted in a tertiary-care university hospital in Madrid, Spain, integrated within the Spanish public healthcare network and providing specialized outpatient care for adults with end-stage kidney disease. The study followed the STROBE recommendations for observational research. This exploratory design was chosen to estimate effect sizes and characterize potential metabolic and gender-related patterns in psychological resilience among adults receiving maintenance HD.

Eligible participants were adults aged ≥ 18 years who had been receiving thrice-weekly HD for at least 12 months. Exclusion criteria were: (1) cognitive or sensory impairment preventing questionnaire completion, as determined through routine clinical assessment by the treating nephrology team based on functional communication ability and capacity to understand study procedures; (2) language or literacy barriers hindering comprehension of study materials; (3) refusal to participate; and (4) inability to complete assessments due to transfer, transplantation, treatment withdrawal, or death.

**Figure 1:**
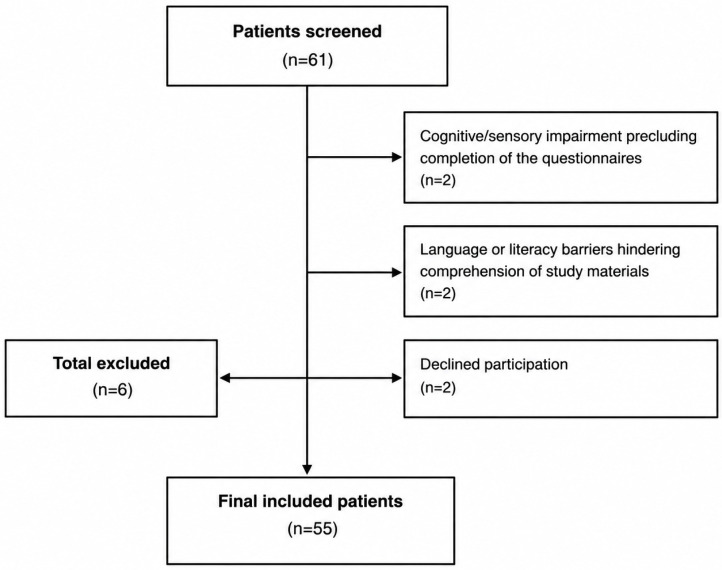
Flow chart of participant inclusion and exclusion.

A total of 61 patients were screened, of whom 55 met all inclusion criteria and completed the study procedures. Reasons for exclusion included cognitive or sensory impairment (n = 2), language or literacy barriers (n = 2), and refusal to participate (n = 2). No additional losses occurred after enrollment. The participant flow is shown in Figure 1.

Dialysis care protocols, laboratory procedures, and assessment timing were standardized across participants to ensure methodological consistency and minimize variability related to treatment conditions.

### 2.2. Data Collection

Data were collected consecutively during routine hemodialysis sessions. Before enrollment, participants received standardized verbal and written information about the study and provided written informed consent.

Psychological assessments were administered before the start of the hemodialysis session using standardized instructions provided by a trained member of the research team. This timing was chosen to minimize the potential influence of intradialytic symptoms, treatment-related fatigue, and post-dialysis emotional fluctuations on questionnaire responses.

Clinical and biochemical data were obtained from the electronic health record. To ensure temporal alignment between psychological and laboratory measures, biochemical parameters were extracted from the monthly pre-dialysis assessment closest to questionnaire administration. Sociodemographic variables, including age and self-identified gender, were collected through a brief self-administered questionnaire, whereas clinical variables (dialysis vintage, diabetes mellitus, and cardiovascular disease) were retrieved from medical records. Gender was assessed through self-report rather than clinical records to capture gender as a self-identified construct.

Completed questionnaires and study data were reviewed for completeness and consistency before statistical analysis. No missing data were observed for variables included in the final analyses.

#### 2.2.1. Sociodemographic and Treatment-Related Variables

Sociodemographic variables included age, self-reported gender, marital status, and educational level, collected through a brief self-administered questionnaire completed before the psychological assessment. Treatment-related variables, including hemodialysis sessions per week, session duration, and dialysis vintage, were obtained from the electronic health record. Sociodemographic and treatment-related variables were used primarily for descriptive characterization and covariate adjustment in the regression models when appropriate. Detailed variable coding and classification are provided in Table 1.

#### 2.2.2. Psychological Assessment—Resilience

Psychological resilience was assessed using the 14-item Resilience Scale (RS-14), originally developed by Wagnild and Young [23] and validated for the Spanish population by Sánchez-Teruel and Robles-Bello [24]. The Spanish version has demonstrated adequate internal consistency (α = 0.79) and is widely used in individuals with chronic illness, supporting its suitability for HD populations.

The RS-14 captures the five core dimensions proposed in the original theoretical model—perseverance, self-reliance, equanimity, meaningfulness, and existential aloneness—providing a concise multidimensional representation of psychological adaptation. Each item is rated on a 7-point Likert scale (1 = strongly disagree to 7 = strongly agree), yielding a total score from 14 to 98, with higher scores indicating greater resilience.

Consistent with prior research and to maintain focus on the metabolic–emotional axis hypothesis, analyses were based on the RS-14 total score, which functions empirically as a continuous variable due to its broad range and summative structure. Participants completed the questionnaire in a quiet area of the dialysis unit immediately before the HD session to minimize fatigue-related or intradialytic emotional variability. Study personnel provided standardized instructions and ensured that responses remained voluntary and confidential.

Completed questionnaires were checked immediately for missing or unclear responses, and data were entered into the study database using a double-entry validation system. To facilitate descriptive interpretation, resilience levels were classified using the cut-offs proposed by Wagnild and Young (1993) [23]: very low (14–56), low (57–64), moderately low (65–73), moderate (74–81), moderately high (82–90), and high (91–98).

The RS-14 has been widely used in chronic disease settings, reinforcing its validity as a global indicator of psychological resilience and its clinical relevance for individuals undergoing long-term hemodialysis.

#### 2.2.3. Biochemical Parameters

Biochemical measurements were obtained from routine pre-dialysis blood samples collected before the mid-week hemodialysis session and analyzed at the Central Defense Hospital “Gómez Ulla” laboratory (Madrid, Spain). The primary biochemical predictors included β_2_-microglobulin, serum albumin, calcium, and 25-hydroxyvitamin D, selected based on their biological relevance to inflammatory, nutritional, and mineral–metabolic pathways potentially related to psychological resilience. Additional biochemical variables were included for descriptive characterization of the cohort. All biochemical parameters were analyzed as continuous variables in their original measurement scales. Detailed laboratory specifications and biochemical classifications are provided in Table 2.

#### 2.2.4. Classification of Variables

The RS-14 total score was analyzed as a continuous variable. Biochemical parameters were analyzed in their original measurement scales, and adjusted regression models incorporated age, dialysis vintage, gender, diabetes mellitus, and cardiovascular disease as clinically relevant covariates. Detailed variable coding, classification, and analytical specifications are provided in Table 3, Table 4 and Table 5.

### 2.3. Statistical Analysis

Statistical analyses were performed using SPSS version 29.0 (IBM Corp., Armonk, NY, USA) and R version 4.3.1 (R Foundation for Statistical Computing, Vienna, Austria). Continuous variables were summarized as means ± standard deviations or medians and interquartile ranges, depending on distributional properties assessed using the Shapiro–Wilk test, whereas categorical variables were expressed as frequencies and percentages. Group comparisons by gender were conducted using independent-samples t tests (Welch correction when appropriate) or Mann–Whitney U tests. Effect sizes were estimated using Cohen’s d and partial eta squared (η^2^_p_).

Spearman correlation analyses were performed to examine bivariate associations between resilience and biochemical parameters. False discovery rate correction was applied using the Benjamini–Hochberg procedure (q = 0.10).

Multivariable linear regression models with HC3 robust standard errors were used to evaluate the association between biochemical markers and psychological resilience (RS-14 total score). Adjusted models included age, gender, dialysis vintage, diabetes mellitus, and cardiovascular disease as clinically relevant covariates. Two interaction terms (Gender × β_2_-microglobulin and Gender × albumin) were specified a priori. Bootstrap resampling (5000 iterations) and penalized regression approaches were performed as sensitivity analyses to assess model stability.

All statistical tests were two-sided, and *p* < 0.05 was considered statistically significant. Analyses were conducted in accordance with STROBE recommendations for observational studies.

### 2.4. Ethical Considerations

The study protocol was reviewed and approved by the Research Ethics Committee of the Central Defense Hospital “Gómez Ulla” (Madrid, Spain; approval number 3/23, issued on 31 March 2023)**.** All procedures complied with internationally recognized ethical standards, including the Declaration of Helsinki [25], the Spanish Biomedical Research Act 14/2007 [26], and the General Data Protection Regulation governing the protection of personal data within the European Union. Additional guidance was drawn from the International Ethical Guidelines for Health-Related Research Involving Humans issued by the Council for International Organizations of Medical Sciences [27].

All participants provided written informed consent after receiving detailed verbal and written information about the aims, procedures, and confidentiality safeguards of the study. Data anonymity and secure handling were ensured in full accordance with European and Spanish regulatory frameworks. No intervention beyond routine clinical care was performed, and no financial incentives or compensations were offered. The study adhered strictly to the ethical, legal, and participant-protection standards applicable to research involving human subjects.

## 3. Results

### 3.1. Characteristics of the Study Population

The study included patients undergoing maintenance hemodialysis with a predominance of men over women. Men were modestly older than women, whereas the prevalence of diabetes and cardiovascular disease was comparable between genders.

Women exhibited significantly lower levels of albumin, calcium, and 25-hydroxyvitamin D than men, indicating a less favorable nutritional and mineral profile. No relevant gender differences were observed in hemoglobin, hematocrit, phosphorus, potassium, or iron. β_2_-microglobulin concentrations were moderately higher in women, although this difference did not reach statistical significance.

With respect to psychological status, women showed significantly lower resilience levels than men. The mean psychological resilience score (RS-14) in the overall sample was 74.02 ± 13.98. According to the cut-off values proposed by Wagnild and Young (low: ≤60; moderate: 61–81; high: ≥82), 18.18% of participants exhibited low resilience, 40.00% moderate resilience, and 41.82% high resilience, indicating a moderate level of psychological resilience at the group level with substantial inter-individual variability.

When stratified by gender, 54.29% of men were classified within the high resilience range compared with 20.00% of women, whereas 25.00% of women and 14.29% of men fell within the low resilience category.

A detailed summary of baseline characteristics is provided in Table 6.

### 3.2. Bivariate Associations Between Resilience and Biochemical Markers

Bivariate correlation analyses identified β_2_-microglobulin as the only biochemical marker significantly associated with psychological resilience in the overall sample. Specifically, higher β_2_-microglobulin levels were associated with lower resilience scores, and this association remained statistically significant after correction for multiple testing using the false discovery rate procedure.

**Figure 2:**
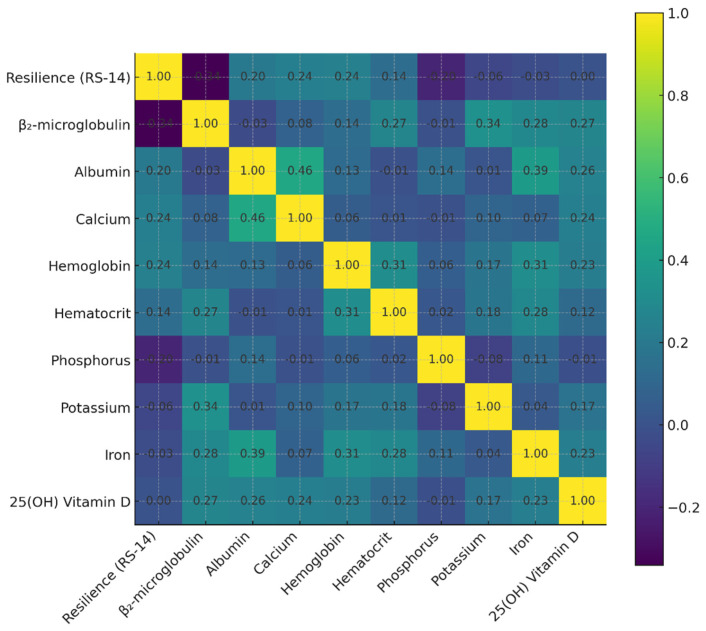
Heatmap of pairwise Spearman correlation coefficients (ρ) between psychological resilience (RS-14) and the assessed biochemical parameters, including β_2_-microglobulin, albumin, calcium, hemoglobin, hematocrit, phosphorus, potassium, iron, and 25-hydroxyvitamin D. Color intensity reflects the magnitude and direction of the correlations. In the overall sample, higher β_2_-microglobulin concentrations were descriptively associated with lower resilience scores.

In contrast, no significant bivariate associations were observed between resilience and albumin, calcium, hemoglobin, hematocrit, phosphorus, potassium, iron, or 25-hydroxyvitamin D after FDR adjustment, indicating that the resilience–biochemistry relationship at the univariate level was primarily characterized by the association with β_2_-microglobulin, whereas no significant relationships were observed for nutritional, mineral, or anemia-related parameters (Table 7, Figure 2).

When analyses were stratified by gender, an inverse association between β_2_-microglobulin and resilience was descriptively observed in men, whereas no statistically significant association was detected in women. However, these findings should be interpreted cautiously given the absence of a statistically significant Gender × Biomarker interaction in the multivariable analyses.

**Figure 3:**
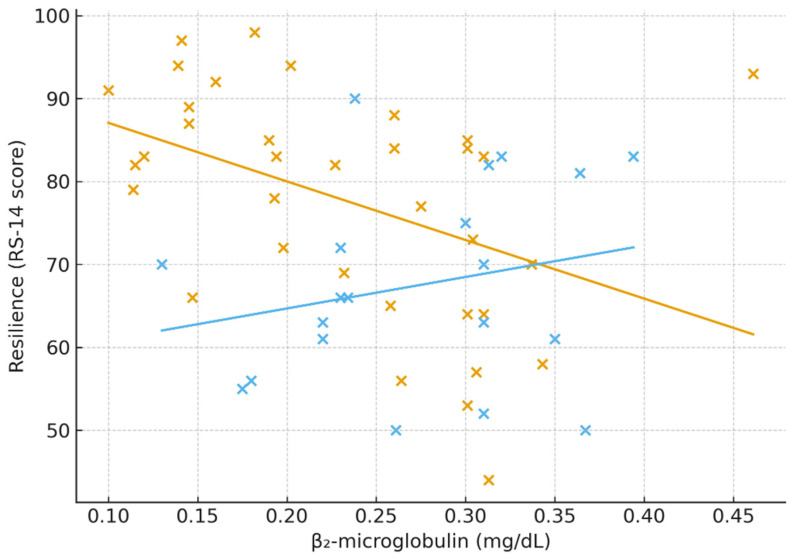
Gender-stratified scatterplot illustrating the association between β_2_-microglobulin concentrations and psychological resilience (RS-14 total score). Blue symbols and regression line represent men, whereas orange symbols and regression line represent women. Fitted regression lines with 95% confidence bands are shown for visualization purposes. In the overall sample, higher β_2_-microglobulin levels were associated with lower resilience scores (Spearman’s ρ = −0.24, *p* = 0.041). However, the β_2_-microglobulin × gender interaction term did not reach statistical significance in the adjusted multivariable model (interaction = 0.162). Therefore, this figure should be interpreted as an exploratory visualization of descriptive gender-related patterns rather than confirmatory evidence of effect modification.

This descriptive pattern is illustrated in the gender-stratified interaction plot (Figure 3). However, given the limited sample size and the absence of a statistically significant interaction term in subsequent multivariable analyses, these gender-stratified findings should be interpreted as exploratory.

Overall, the bivariate analyses consistently indicate that β_2_-microglobulin represents the primary biochemical correlate of psychological resilience in this hemodialysis cohort.

The complete numerical results of the fully adjusted multivariable regression model, including unstandardized regression coefficients, HC3 robust standard errors, 95% confidence intervals, and p-values for all predictors and covariates, are provided in Table 8.

### 3.3. Multivariable Models and Gender Interactions

Multivariable linear regression models were constructed to examine the independent association between β_2_-microglobulin and psychological resilience after adjustment for relevant demographic and clinical covariates. Resilience (RS-14 total score) was entered as the dependent variable in all analyses. Predictors included β_2_-microglobulin, gender (women vs. men), age, diabetes mellitus, and cardiovascular disease. Robust HC3 standard errors were applied to account for potential heteroscedasticity.

In Model 1, which included only main effects, higher β_2_-microglobulin levels showed a consistent inverse association with resilience; however, this relationship did not reach statistical significance after multivariable adjustment. Gender was also inversely associated with resilience, indicating lower scores in women compared with men, although this effect did not reach statistical significance in this initial model. Age, diabetes, and cardiovascular disease were not independently associated with resilience.

In Model 2, the interaction term between β_2_-microglobulin and gender was introduced to formally test effect modification. The inclusion of this term slightly increased the explained variance of the model (ΔR^2^ = 0.06). However, the β_2_-microglobulin × gender interaction did not reach statistical significance, indicating that, within the limits of the sample size, there was no robust statistical evidence that the association between β_2_-microglobulin and resilience differed by gender.

Notably, after inclusion of the interaction term, female gender emerged as an independent predictor of lower resilience, with women exhibiting significantly lower resilience scores than men after full adjustment. This change relative to Model 1 likely reflects the redistribution of explained variance following the inclusion of the interaction term.

**Figure 4:**
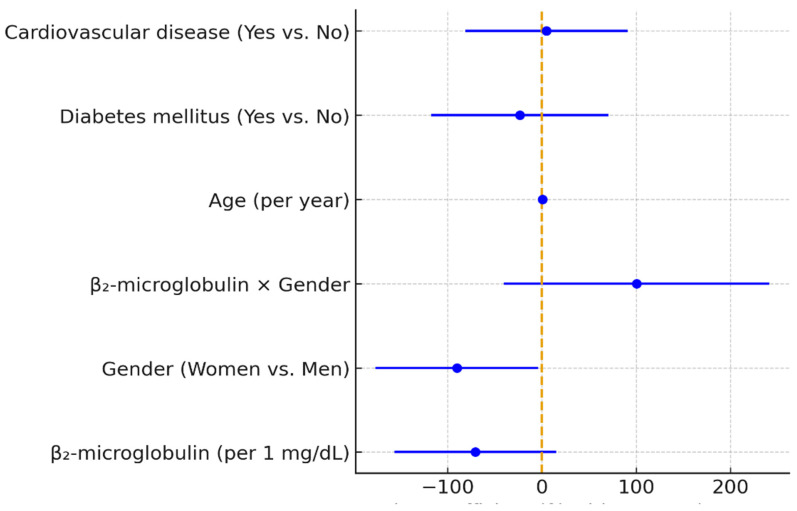
Unstandardized regression coefficients (β) with 95% confidence intervals from the fully adjusted model examining the association between resilience and β_2_-microglobulin, gender, and their interaction, adjusted for age, diabetes mellitus, and cardiovascular disease. The vertical dashed line represents the null effect (β = 0). Estimates were obtained using ordinary least squares regression with HC3 robust standard errors.

Overall, these multivariable findings indicate that the inverse relationship between β_2_-microglobulin and resilience observed in the bivariate analyses is partially attenuated after clinical adjustment, whereas gender remains independently associated with resilience. The absence of a statistically significant interaction indicates that the apparent gender-related differences observed in the stratified and graphical analyses should be interpreted as exploratory and descriptive rather than as evidence of true effect modification. (Table 9, Figure 4).

Model performance was modest. In Model 1, the coefficient of determination was R^2^ = 0.20, with an adjusted R^2^ of 0.12, indicating that approximately 20% of the variance in resilience was explained by the main effects included in the model, with a more conservative estimate of 12% after adjustment for the number of predictors.

In Model 2, which incorporated the interaction term, the explained variance increased to R^2^ = 0.26, with an adjusted R^2^ of 0.17. The inclusion of the interaction resulted in a ΔR^2^ of +0.06, reflecting a 6% increase in explained variance compared with Model 1, although this incremental contribution did not reach statistical significance.

## 4. Discussion

This exploratory study investigated the relationship between psychological resilience and routinely monitored biochemical parameters in patients receiving maintenance hemodialysis, with particular attention to potential gender-related differences. Three principal findings emerged. First, higher serum β_2_-microglobulin concentrations were associated with lower resilience at the bivariate level, although this relationship was attenuated after multivariable adjustment. Second, women exhibited significantly lower resilience scores than men and a less favorable biochemical profile characterized by lower albumin, calcium, and 25-hydroxyvitamin D concentrations. Third, despite descriptive differences observed in stratified analyses, no significant Biomarker × Gender interactions were identified, suggesting that gender did not substantially modify the associations between biochemical markers and resilience in the adjusted models.

The most important finding of the present study is the identification of lower resilience among women receiving maintenance hemodialysis. Resilience is increasingly recognized as a clinically relevant determinant of adaptation to chronic kidney disease and dialysis treatment. Recent evidence suggests that resilience is associated with lower emotional distress, greater treatment engagement, improved coping strategies, and better health-related quality of life in patients undergoing renal replacement therapy [3,4,5,6,7,8,9,10]. Shahin et al. [3] emphasized resilience as a dynamic and potentially modifiable construct across the spectrum of advanced kidney disease, while García-Martínez et al. [10] identified psychosocial resources as major determinants of resilience in long-term hemodialysis patients. Similarly, Wang et al. [5] demonstrated longitudinal associations between resilience, family resilience, and social support, reinforcing the concept that resilience represents an adaptive process rather than a fixed personality trait. The present findings extend this literature by suggesting that gender-related differences remain evident even within a relatively homogeneous clinical population receiving the same treatment modality.

The mechanisms underlying these differences are likely multifactorial. Previous studies have consistently reported higher rates of depression, anxiety, and psychological burden among women undergoing maintenance hemodialysis [19,28,29,30,31,32,33,34]. These differences may reflect unequal exposure to psychosocial stressors, caregiving responsibilities, illness perceptions, and coping resources. In addition, biological differences in immune and inflammatory regulation have been proposed as potential contributors to sex-related variation in symptom burden and psychological outcomes [20,21,22]. Although the present study was not designed to investigate causal pathways, the coexistence of lower resilience and a less favorable biochemical profile among women suggests that biological and psychosocial determinants of adaptation may interact in complex ways. Importantly, the observed difference may have clinical relevance beyond statistical significance because resilience has been associated with treatment adherence, emotional adjustment, and quality of life outcomes in dialysis populations [4,8,10].

Another notable finding was the inverse relationship between resilience and β_2_-microglobulin. β_2_-microglobulin is widely recognized as a marker of middle-molecule retention, dialysis-related biological stress, and chronic inflammatory burden [14,15,16,17,31]. Elevated concentrations have consistently been associated with adverse outcomes, including cardiovascular complications, hospitalization, and mortality [14,15,16,17,31]. However, previous studies have primarily focused on hard clinical outcomes, and little attention has been paid to potential associations with adaptive psychological processes. To our knowledge, few studies have explored the relationship between resilience and β_2_-microglobulin in maintenance hemodialysis populations. Therefore, the present findings contribute novel evidence suggesting that biological burden and psychological adaptation may be interconnected dimensions of the patient experience.

Several mechanisms may explain this association. Chronic inflammation and uremic toxin accumulation have been linked to depressive symptoms, fatigue, cognitive dysfunction, and impaired quality of life among patients receiving hemodialysis [19,32,33,34,35]. These factors may reduce psychological resources available for adaptation and increase vulnerability to emotional distress. Consequently, the observed inverse association between β_2_-microglobulin and resilience is biologically plausible and consistent with contemporary models linking inflammatory burden to psychological outcomes. Nevertheless, the disappearance of statistical significance after multivariable adjustment suggests that β_2_-microglobulin is unlikely to act as an independent determinant of resilience. Instead, it may represent a surrogate marker of broader biological and clinical processes that overlap with other determinants of psychological adaptation. This distinction is important because it highlights the difference between statistical association and independent explanatory value.

Women in the present cohort also exhibited lower albumin, calcium, and 25-hydroxyvitamin D concentrations. Albumin is widely regarded as an integrative marker of nutritional status and chronic inflammation and has previously been associated with depressive symptoms, fatigue, and poorer quality of life in kidney disease populations [35,36,37]. Likewise, abnormalities in mineral metabolism and vitamin D deficiency have been linked to emotional distress and adverse patient-reported outcomes in chronic kidney disease [18,19]. However, none of these biomarkers demonstrated independent associations with resilience in the adjusted analyses. These findings suggest that resilience may not be adequately explained by isolated biochemical abnormalities and instead reflects a multidimensional construct shaped by biological, psychological, and social influences. This interpretation is consistent with contemporary conceptual models that view resilience as the result of complex interactions between individual, interpersonal, and disease-related factors [3,5,9,12].

From a clinical perspective, the present findings reinforce the importance of incorporating psychosocial assessment into routine hemodialysis care. Although the evaluated biomarkers cannot currently be considered predictive indicators of resilience, the coexistence of lower resilience and less favorable biological profiles highlights the need for a comprehensive biopsychosocial approach to patient management. Routine assessment of resilience may facilitate the identification of patients at increased risk of emotional maladaptation and may help guide supportive interventions aimed at improving coping, treatment engagement, and quality of life. Such an approach is aligned with contemporary recommendations emphasizing holistic and patient-centered nephrology care [13].

The statistical and clinical implications of these findings warrant careful consideration. While several associations reached statistical significance, the attenuation of effects after multivariable adjustment indicates that resilience is influenced by multiple interacting determinants rather than by individual biochemical markers in isolation. Consequently, the principal clinical contribution of this study lies not in identifying specific biomarkers as predictors of resilience, but in supporting a multidimensional understanding of psychological adaptation in patients receiving maintenance hemodialysis. This perspective may be particularly relevant for the development of future interventions targeting both emotional well-being and overall patient-centered outcomes.

Taken together, these findings contribute to the growing body of evidence recognizing resilience as a clinically meaningful construct in chronic kidney disease. Future multicenter longitudinal studies are needed to clarify temporal relationships between biological burden, inflammatory processes, and resilience and to determine whether interventions designed to strengthen resilience can improve both patient-reported and clinical outcomes in maintenance hemodialysis populations.

### 4.1. Clinical Implications and Future Directions

The present findings suggest that metabolic–inflammatory disturbances may help identify hemodialysis patients with greater emotional vulnerability. Exploratory stratified analyses indicated a possible inverse association between β_2_-microglobulin and resilience, particularly among men; however, in the absence of statistically significant Gender × Biomarker interaction effects, these findings should be interpreted cautiously and considered hypothesis-generating. Previous studies linking inflammation, uremic toxins, and immune-related abnormalities with mood symptoms and poorer clinical stability in hemodialysis populations further support the potential relevance of closer psychosocial monitoring in vulnerable patient subgroups [32,33].

Although women exhibited lower serum albumin levels than men, albumin did not emerge as an independent predictor of resilience in adjusted models and should therefore be interpreted primarily as a descriptive marker of nutritional–inflammatory vulnerability rather than a direct explanatory factor of psychological resilience. This interpretation is consistent with previous studies linking hypoalbuminemia to depressive symptoms, anxiety, and poorer quality of life in chronic kidney disease populations [35,36,37,38]. These findings support the potential value of integrating nutritional assessment and dietary support into routine psychosocial care in hemodialysis settings.

Across genders, psychosocial interventions have been associated with improved mental health and resilience in hemodialysis populations [4,6,7,8,9,12]. Integrating metabolic assessment into psychosocial care may help identify patients with greater emotional vulnerability.

Taken together, these findings support a more integrated approach in hemodialysis care combining biochemical monitoring and psychosocial assessment to identify patients at greater emotional vulnerability. Future multicenter longitudinal studies are needed to clarify the clinical relevance and reproducibility of the observed gender-related patterns.

### 4.2. Strengths and Limitations

This study has several strengths that enhance its clinical relevance and internal validity, including a clinically homogeneous hemodialysis cohort, standardized laboratory analyses performed in a single laboratory, use of the validated RS-14 resilience scale, and a robust statistical strategy incorporating HC3 robust estimators, bootstrap resampling, penalized regression models, and interaction analyses. Adjustment for major clinical confounders further strengthened the interpretability of the findings, while the study addresses a largely unexplored metabolic–emotional dimension of psychological resilience in nephrology.

Several limitations should also be acknowledged. The cross-sectional design precludes causal inference, and the modest sample size—particularly among women—may have limited statistical power, especially for interaction analyses. The single-center setting may also reduce generalizability. In addition, gender was operationalized as a binary self-reported variable, which may not fully capture the psychosocial complexity of gender identity. Residual confounding, self-report bias, and the use of single time-point biochemical measurements should also be considered. Overall, the findings should be interpreted as exploratory and require confirmation in larger multicenter longitudinal studies.

## 5. Conclusions

This exploratory study suggests that routinely monitored metabolic and inflammatory biomarkers may be related to psychological resilience in adults receiving maintenance hemodialysis, although these associations were not independently sustained after multivariable adjustment. Women exhibited lower resilience scores together with a less favorable biochemical profile, supporting the relevance of gender-sensitive assessment in chronic dialysis care.

These findings reinforce the importance of integrating psychosocial and biological dimensions into the clinical evaluation of patients undergoing maintenance hemodialysis and support the hypothesis of a potential metabolic–emotional interface in chronic kidney disease. Further multicenter longitudinal studies with larger samples are needed to clarify the temporal and mechanistic relationships between metabolic dysregulation, inflammation, gender-related factors, and psychological resilience in hemodialysis populations.

## Figures and Tables

**Table 1:** Sociodemographic and Treatment-Related Variables: Definition, Coding, and Analytical Use.

Variable	Source	Type	Coding/Units	Analytical Use	Relevance
Age	Self-administered questionnaire	Continuous	Years	Descriptive, covariate	Core demographic determinant
Gender	Self-administered questionnaire	Nominal dichotomous	0 = man; 1 = woman	Stratification, moderator	Psychosocial and demographic determinant.
Marital status	Self-administered questionnaire	Nominal polytomous	Single/Married/Widowed/Divorced	Descriptive	Proxy of social support
Educational level	Self-administered questionnaire	Ordinal	0–4 (increasing education)	Descriptive	Socioeconomic and health literacy indicator
HD sessions per week	Electronic health record	Discrete quantitative	Sessions/week	Descriptive	Treatment intensity
HD session duration	Electronic health record	Continuous	Minutes	Descriptive	Intradialytic exposure
Dialysis vintage	Electronic health record	Continuous	Months/years	Descriptive, covariate	Chronic RRT exposure

Note. Sociodemographic variables were self-reported. Treatment-related variables were extracted from the electronic health record.

**Table 2:** Laboratory methods, reference ranges, and clinical relevance of biochemical parameters.

Parameter	Analytical Method/Instrument	Units	Reference Range	Clinical Relevance
β_2_-microglobulin	Immunonephelometry (Siemens BN II)	mg/dL	1.0–3.0	Marker of middle-molecule clearance, chronic inflammation, and dialyzer biocompatibility.
Albumin	Bromocresol-green colorimetry (Roche Cobas 8000)	g/dL	3.5–5.0	Reflects nutritional–inflammatory status; hypoalbuminemia predicts morbidity.
Hemoglobin	Automated hematologic spectrophotometry (Sysmex XN-1000)	g/dL	12–16 (men); 11–15 (women)	Index of erythropoietic function and anemia control.
Hematocrit	Calculated from erythrocyte indices (Sysmex XN-1000)	%	36–46 (men); 33–43 (women)	Correlates with oxygen-carrying capacity.
Phosphorus	UV colorimetry with molybdate (Roche Cobas 8000)	mg/dL	2.5–4.5	Key mineral metabolism marker; hyperphosphatemia linked to vascular calcification.
Potassium	Ion-selective electrode potentiometry	mEq/L	3.5–5.5	Essential for electrolyte balance; deviations may influence anxiety and neuromuscular excitability.
Iron	Ferrozine colorimetry (Roche Cobas 8000)	μg/dL	60–160	Reflects circulating iron levels and erythropoietic status; ferritin and transferrin saturation are more appropriate markers of iron stores in hemodialysis patients.
Calcium	Arsenazo III colorimetry (Roche Cobas 8000)	mg/dL	8.5–10.2	Related to neuromuscular excitability and bone mineralization.
25-hydroxyvitamin D [25(OH)D]	Chemiluminescent immunoassay (Abbott Architect i2000SR)	ng/mL	30–100	Low levels associated with inflammation, fatigue, and depressive symptoms.

Note. A ll variables were analyzed as continuous measures. β_2_-microglobulin, phosphorus, and 25-hydroxyvitamin D were analyzed in their original measurement scales without logarithmic transformation, as model assumptions were adequately met using robust HC3 estimators.

**Table 3:** Statistical classification and analytical treatment of the psychological resilience variable (RS-14).

Variable	Statistical Type	Scale	Notes
Resilience (RS-14)	Quasi-continuous (treated as continuous)	Interval	Total score range 14–98. Ordinal items summed into a continuous-acting total; supported by psychometric evidence in chronic illness populations.

Note. The RS-14 was analyzed as a quasi-continuous measure, allowing the use of parametric or robust regression techniques (HC3 with bootstrap).

**Table 4:** Statistical classification, analytical methods, and clinical relevance of biochemical variables.

Variable	Statistical Type	Scale	Units/Method	Notes
β_2_-microglobulin	Continuous	Ratio	mg/dL; immunonephelometry	Marker of inflammation and middle-molecule clearance.
Albumin	Continuous	Ratio	g/dL; bromocresol-green colorimetry	Normal distribution; indicator of nutritional–inflammatory status.
Hemoglobin	Continuous	Ratio	g/dL; automated spectrophotometry	Reflects anemia status and erythropoietic function.
Hematocrit	Continuous	Ratio	%; derived erythrocyte indices	Correlates with oxygen-carrying capacity.
Phosphorus	Continuous	Ratio	mg/dL; UV molybdate colorimetry	Key marker of mineral-bone metabolism.
Potassium	Continuous	Ratio	mEq/L; ion-selective electrode	Reflects electrolyte balance; related to neuromuscular excitability.
Iron	Continuous	Ratio	µg/dL; ferrozine colorimetry	Indicates iron stores; deficiency associated with fatigue and low mood.
Calcium	Continuous	Ratio	mg/dL; arsenazo III colorimetry	Linked to neuromuscular function and bone mineralization.
25-hydroxyvitamin D	Continuous	Ratio	ng/mL; chemiluminescent immunoassay	Associated with inflammation and psychological symptoms.

Note. All biochemical variables were analyzed as continuous measures in their original measurement scale without logarithmic transformation. Robust HC3 estimators were used to account for potential deviations from normality.

**Table 5:** Coding and analytical treatment of demographic and clinical covariates included in regression models.

Variable	Statistical Type	Scale	Coding/Description	Notes
Age	Continuous	Ratio	Years	Used as a continuous covariate.
Gender	Discrete (binary)	Nominal	0 = Men; 1 = Women	Self-reported using three response options (man, woman, other/prefer not to answer); all participants identified as man or woman; primary stratification variable.
Hemodialysis vintage	Continuous/ordinal	Ratio/ordinal	Months; optionally categorized (1–3, 4–6, >6 years)	Continuous for regression; categorical for descriptive reporting.
Diabetes mellitus	Discrete (binary)	Nominal	0 = No; 1 = Yes	Primary comorbidity.
Cardiovascular disease	Discrete (binary)	Nominal	0 = No; 1 = Yes	Secondary comorbidity.

Note. Dichotomous clinical variables were coded as dummy variables (0 = absence, 1 = presence). Hemodialysis vintage was analyzed as a continuous predictor in inferential models.

**Table 6:** Baseline characteristics of the study population, overall and stratified by gender.

Variable	Total	Men	Women	Mean Diff (Men–Women)	95% CI Diff	*p*	d
Age (years)	67.71 (15.15), 95% CI 63.55–71.86	71.09 (13.65), 95% CI 66.20–75.28	61.80 (16.16), 95% CI 54.10–69.61	9.29	1.73–16.86	0.037	0.62
R esilience (RS-14)	74.02 (13.98), 95% CI 70.25–77.79	77.71 (13.78), 95% CI 73.01–82.47	67.50 (12.07), 95% CI 61.96–73.04	10.21	3.07–17.40	0.006	0.79
Albumin (g/dL)	3.34 (1.00), 95% CI 3.07–3.61	3.523 (1.18), 95% CI 3.12–3.91	3.017 (0.45), 95% CI 2.80–3.24	0.506	0.05–0.96	0.028	0.57
β_2_-microglobulin (mg/dL)	2.47 (0.81), 95% CI 2.25–2.69	2.33 (0.84), 95% CI 2.03–2.62	2.73 (0.72), 95% CI 2.39–3.06	−0.40	−0.82–0.02	0.068	−0.51
Hemoglobin (g/dL)	11.63 (1.42), 95% CI 11.24–12.02	11.74 (1.53), 95% CI 11.21–12.28	11.44 (1.22), 95% CI 10.88–11.99	0.303	−0.53–1.14	0.424	0.22
Hematocrit (%)	35.92 (4.30), 95% CI 34.75–37.09	36.03 (4.38), 95% CI 34.55–37.57	35.73 (4.27), 95% CI 33.71–37.75	0.301	−2.02–2.68	0.804	0.07
Phosphorus (mg/dL)	4.51 (1.34), 95% CI 4.16–4.86	4.52 (1.54), 95% CI 4.01–5.03	4.49 (0.93), 95% CI 4.08–4.90	0.03	−0.62–0.68	0.93	0.02
Potassium (mEq/L)	4.87 (0.73), 95% CI 4.68–5.06	4.83 (0.83), 95% CI 4.54–5.12	4.93 (0.52), 95% CI 4.67–5.19	−0.10	−0.44–0.24	0.55	−0.14
Iron (µg/dL)	77.63 (42.74), 95% CI 66.01–89.25	83.60 (50.75), 95% CI 66.08–101.12	67.17 (20.04), 95% CI 57.11–77.23	16.43	−7.05–39.90	0.16	0.39
Calcium (mg/dL)	8.95 (0.69), 95% CI 8.76–9.14	9.131 (0.54), 95% CI 8.95–9.32	8.630 (0.82), 95% CI 8.24–9.02	0.501	0.08–0.92	0.021	0.72
25-hydroxyvitamin D (ng/mL)	24.67 (14.30), 95% CI 21.03–28.31	28.112 (15.74), 95% CI 23.05–33.16	18.639 (8.82), 95% CI 14.78–22.50	9.473	2.75–16.19	0.006	0.74
Diabetes mellitus	36 (65.5%) no/19 (34.5%) yes	25 (71.4%) no/10 (28.6%) yes	11 (55.0%) no/9 (45.0%) yes	—	—	—	—
Cardiovascular disease	35 (63.6%) no/20 (36.4%) yes	20 (57.1%) no/15 (42.9%) yes	15 (75.0%) no/5 (25.0%) yes	—	—	—	—
Hemodialysis vintage	1–3 y: 54.5%, 4–6 y: 29.1%, >6 y: 16.4%	54.3%/31.4%/14.3%	55%/25%/20%	—	—	—	—

Note. Continuous variables are reported as Mean (SD) with 95% confidence intervals (CI). Categorical variables are reported as n (%). Between-group comparisons include mean differences (continuous variables only), 95% CI, *p*-values, and Cohen’s d.

**Table 7:** Spearman correlations between resilience (RS-14) and biochemical markers in the total cohort.

Biochemical Marker	Spearman ρ	95% CI	*p*-Value	FDR-Adjusted *p*
β_2_-microglobulin (mg/dL)	−0.24	−0.47 to −0.01	0.041	0.041
Albumin (g/dL)	+0.20	−0.07 to +0.44	0.14	0.21
Calcium (mg/dL)	+0.24	−0.03 to +0.47	0.09	0.18
Hemoglobin (g/dL)	−0.14	−0.39 to +0.14	0.32	0.41
Hematocrit (%)	−0.14	−0.39 to +0.14	0.32	0.41
Phosphorus (mg/dL)	−0.10	−0.37 to +0.18	0.48	0.53
Potassium (mEq/L)	−0.06	−0.34 to +0.22	0.66	0.66
Iron (µg/dL)	−0.03	−0.31 to +0.25	0.82	0.82
25-hydroxyvitamin D (ng/mL)	0.00	−0.28 to +0.28	1.00	1.00

Note. Spearman correlations. Confidence intervals computed using Fisher’s z approximation. FDR-adjusted *p*-values based on Benjamini–Hochberg procedure (q = 0.10).

**Table 8:** Fully adjusted multivariable linear regression model for psychological resilience using HC3 robust standard errors. Fully adjusted multivariable linear regression model for psychological resilience (HC3 robust estimates).

Predictor	β (Unstandardized)	HC3 SE	95% CI Lower	95% CI Upper	*p*-Value
β_2_-microglobulin (per 1 mg/dL)	−7.06	4.68	−15.64	1.52	0.107
Gender (women vs. men)	−9.01	4.36	−17.65	−0.38	0.041
β_2_-microglobulin × Gender	10.05	7.06	−4.05	24.14	0.162
Age (per year)	0.05	0.14	−0.24	0.34	0.723
Diabetes mellitus (yes vs. no)	−2.35	4.79	−11.75	7.05	0.624
Cardiovascular disease (yes vs. no)	0.49	4.38	−8.12	9.10	0.912

Model performance: R^2^ = 0.26; Adjusted R^2^ = 0.17. ΔR^2^ (interaction effect) = +0.06. Outcome: Psychological resilience (RS-14 total score). Method: Ordinary least squares regression with heteroscedasticity-consistent HC3 robust standard errors.

**Table 9:** Multivariable linear regression models for resilience and gender interaction (n = 55).

Predictor	Model 1 β (95% CI)	*p*-Value	Model 2 β (95% CI)	*p*-Value
β_2_-microglobulin (per 1 mg/dL)	−4.42 (−9.92 to 1.08)	0.115	−7.06 (−15.64 to 1.52)	0.107
Gender (Women vs. Men)	−6.96 (−15.55 to 1.62)	0.112	−9.01 (−17.65 to −0.38)	0.041
β_2_-microglobulin × Gender	—	—	10.05 (−4.05 to 24.14)	0.162
Age (per year)	0.08 (−0.20 to 0.35)	0.583	0.05 (−0.24 to 0.34)	0.723
Diabetes mellitus (Yes vs. No)	−4.55 (−12.39 to 3.28)	0.255	−2.35 (−11.75 to 7.05)	0.624
Cardiovascular disease (Yes vs. No)	0.29 (−8.10 to 8.68)	0.946	0.49 (−8.12 to 9.10)	0.912

Outcome: Resilience (RS-14 total score). Predictors: β_2_-microglobulin (per 1 mg/dL), gender (women vs. men), age, diabetes mellitus, and cardiovascular disease. Model 2 includes the interaction term β_2_-microglobulin × gender. Estimates are unstandardized β coefficients with 95% confidence intervals (CI). Robust HC3 standard errors were used.

## Data Availability

The datasets generated and analyzed during the current study are not publicly available due to the presence of sensitive clinical information that could potentially compromise participant confidentiality but are available from the corresponding author on reasonable request.

## Abbreviations

B Ca	Bias-Corrected and Accelerated
BMC	BioMed Central
BN II	Siemens BN II Analyzer
CI	Confidence Interval
CIOMS	Council for International Organizations of Medical Sciences
CKD	Chronic Kidney Disease
ESKD	End-Stage Kidney Disease
EU	European Union
FDR	False Discovery Rate
HC3	Heteroscedasticity-Consistent Standard Error Type 3
HD	Hemodialysis
IBM	IBM SPSS Statistics
ISO	International Organization for Standardization
KDIGO	Kidney Disease: Improving Global Outcomes
R^2^	Coefficient of Determination
RS-14	Resilience Scale–14
SD	Standard Deviation
SEQC-ML	Spanish Society of Laboratory Medicine
STROBE	Strengthening the Reporting of Observational Studies in Epidemiology
UV	Ultraviolet (UV Colorimetry)
VIF	Variance Inflation Factor
XN-1000	Sysmex XN-1000 Hematology Analyzer
